# Cumulative burden of diabetes-related complications and health-related quality of life in primary care: a cross-sectional study from Mexico

**DOI:** 10.3389/fcdhc.2026.1773692

**Published:** 2026-03-12

**Authors:** Ruben Silva-Tinoco, Lilia Castillo-Martínez, Ana Galindez-Fuentes, Alejandro Avalos-Bracho, Edward W Gregg, Teresa Cuatecontzi-Xochitiotzi, Christian Hinojosa-Segura, María Fernanda Bernal-Ceballos

**Affiliations:** 1 Unidad de Atención a la Salud, Servicios Públicos de Salud del Instituto Mexicano del Seguro Social para el Bienestar, Mexico City, Mexico; 2 Department of Clinical Nutrition, Instituto Nacional de Ciencias Médicas y Nutrición Salvador Zubirán, Mexico City, Mexico; 3 Clínica Especializada en el Manejo de la Diabetes en la Ciudad de México, Servicios Públicos de Salud del Instituto Mexicano del Seguro Social para el Bienestar, Mexico City, Mexico; 4 Royal College of Surgeons in Ireland (RCSI) University of Medicine and Health Sciences, Dublin, Ireland; 5 Imperial College London, London, United Kingdom

**Keywords:** diabetes-related complications, EQ-5D-5L, health-related quality of life, primary care, type 2 diabetes

## Abstract

**Background:**

Understanding the coexistence of diabetes-related complications and their impact on quality of life can help guide healthcare strategies.

**Aim:**

The purpose of this study was to assess the prevalence and coexistence of diabetes-related complications and their association with health-related quality of life (HRQoL) in patients with type 2 diabetes (T2D).

**Methods:**

We conducted a cross-sectional study of 1,703 patients with T2D receiving standard care in primary healthcare units in Mexico. Diabetes-related complications assessed included peripheral neuropathy, retinopathy, chronic kidney disease, cardiovascular disease, and amputation history. HRQoL was measured using the EuroQol 5-Dimension 5-Level questionnaire. Multivariable regression analyses were used to adjust for confounders and assess associations with HRQoL.

**Results:**

Complications were present in 70.5% of patients, predominantly peripheral neuropathy. The most affected HRQoL dimensions were pain/discomfort (53.8%) and anxiety/depression (53.4%), followed by mobility (34.2%), self-care (34%), and usual activities (24.2%). Multivariable analysis showed female sex was independently associated with a higher likelihoodof reporting problems across all dimensions. Having at least one complication was also associated with impairment, except for anxiety/depression, where risk increased with two or more complications. Hypertension and use of insulin were associated with a higher risk of problems in mobility, selfcare, and usual activities. Longer diabetes duration and use of insulin were risk factors for pain/discomfort, while higher body mass index was associated with anxiety/depression.

**Conclusion:**

Diabetes-related complications are highly prevalent and affect HRQoL domains in primary care, with a greater burden as complications accumulate.

## 1 Introduction

Diabetes is one of the fastest-growing global health challenges of the 21st century, with an estimated 589 million adults living with the condition in 2024 (1). A major contributor to the overall burden of diabetes is the broad spectrum of associated complications, which have a profound impact on healthcare needs and costs, disability, health-related quality of life (HRQoL), and premature mortality.

Despite remarkable progress in the management of type 2 diabetes (T2D) and other related chronic conditions, including obesity and cardiovascular diseases, significant challenges persist in achieving optimal care targets and improving clinical outcomes. The patient-centered care model has emerged as a key strategy to address these gaps by actively involving individuals in treatment decisions that reflect their preferences, values, and needs. This approach has been associated with improved health behaviors, more appropriate use of healthcare services, and reduced symptom burden, and is now recognized as a core component of contemporary diabetes care in international guidelines (2).

In this context, expert consensus has emphasized the importance of incorporating patient-reported outcomes (PROs) into both clinical practice and research. These measures, which include aspects such as well-being, daily functioning, and HRQoL, provide valuable insights into the patient experience and complement traditional biomedical indicators (3).

In chronic conditions such as diabetes, which require ongoing clinical care and individual self-management, the preservation of patients’ HRQoL is recognized as a core objective of treatment (2, 4–6). However, in Latin America, evidence on the coexistence of diabetes-related complications and their impact on HRQoL remains limited. Biological, psychosocial, contextual, and environmental factors specific to this region may influence the development of complications and individuals’ subjective health perceptions (7).

Although the clinical consequences of diabetes-related complications are well documented, their impact on HRQoL, particularly in the early stages of the disease and in primary care contexts, is less well understood (8, 9). Primary care plays a pivotal role in the detection and management of chronic diseases. However, comprehensive evaluations of diabetes-related complications and their associations with specific HRQoL domains in this setting remain scarce. This is especially relevant in countries such as Mexico, where the burden of diabetes is high; however, evidence regarding HRQoL assessment in primary care populations is limited.

Given these gaps in the literature, a more thorough understanding is needed to characterize the burden and coexistence of diabetes-related complications and their association with HRQoL in real-world primary-care settings. This study aimed to examine the prevalence and overlap of diabetes-related complications and assess their impact on specific HRQoL domains in adults with T2D receiving routine care in primary care units in Mexico.

## 2 Methods

### 2.1 Study design

This cross-sectional study used prospectively collected primary data. The DIABetes EMPowerment and Improvement of Care (DIABEMPIC) program is a quality improvement initiative designed to enhance health outcomes among individuals living with T2D in public primary care settings in Mexico City. A Clinic Specialized in Diabetes Management (CEDM), which is part of the primary care stewardship, was established to implement the health interventions included in the initiative. Since its creation in 2017, the clinic has contributed to a better understanding of the needs and opportunities for improving diabetes care in primary healthcare settings through systematic documentation of population characteristics and program effectiveness (10–12).

### 2.2 Settings and subjects

The components of the program and participant recruitment procedures have been described in detail elsewhere (12). Briefly, individuals were referred from 18 primary care units to the CEDM for a comprehensive, interdisciplinary diabetes care program. The 18 participating primary care units were included within the framework of the DIABEMPIC program and were geographically proximate to facilitate patient follow-up and adherence to program activities. The participants were consecutively enrolled between July 2017 and January 2024. Exclusion criteria included acute conditions requiring immediate hospital care, such as severe hyperglycemia requiring hospital management, newly diagnosed foot ulcer, acute coronary syndrome, recent stroke, and kidney disease requiring renal replacement therapy, as well as pregnancy and any diabetes diagnosis other than T2D. The study was approved by the Ethics Committee of the Mexico City Ministry of Health (609-01-01-18), and all participants provided both verbal and written informed consent. This study was registered at ClinicalTrials.gov (NCT04245267) and followed STROBE guidelines.

### 2.3 Assessments of participants

A cornerstone component of the program is the systematic screening and detection of diabetes-related complications to enable timely therapeutic interventions.

Sociodemographic data and clinical characteristics, including comorbidities and current pharmacological treatments, were obtained from medical records and corroborated through medical interviews and physical examinations. Biochemical parameters were updated at the time of enrollment. The status of diabetes-related complications, including diabetic retinopathy, chronic kidney disease (CKD), peripheral diabetic neuropathy, cardiovascular disease (CVD), and history of amputation, was also updated through standardized clinical assessments (13).

Diabetic retinopathy was assessed by an ophthalmologist using a mydriatic camera (Canon CF-1, Japan). CKD was defined by estimated glomerular filtration rate values of <60 mL/min/1.73 m^2^ and/or albuminuria as indicated by an albumin/creatinine ratio ≥30 mg/g. Peripheral neuropathy was defined as the presence of loss of protective sensation identified through pressure perception and vibration perception tests, as well as compatible symptomatology. The absence of pressure perception with the 10 g Semmes-Weinstein monofilament and/or the absence of vibration perception (128 Hz tuning fork) in either foot was considered a loss of protective sensation.

HRQoL was assessed using the EuroQol 5-Dimensional 5-Level questionnaire (EQ-5D-5L), which includes a brief descriptive classification system covering five health dimensions and a visual analog scale (EQ VAS). The EQ-5D-5L questionnaire is one of the most widely used preference-based measures of HRQoL across various health conditions worldwide. It has demonstrated good validity and reliability in individuals with T2D and is commonly applied in both clinical and population-based studies (14, 15). Its simplicity, short administration time, and ability to reflect the impact of chronic conditions on daily functioning make it particularly suitable for use in primary care settings. Moreover, it facilitates the identification of the health domains most affected by chronic diseases, such as T2D. Additionally, it adds value by having been specifically validated for use in the Mexican population (16).

The questionnaire required patients to self-report their health status across the following dimensions: mobility, self-care, usual activities, pain/discomfort, and anxiety/depression. Each dimension was rated using a five-level severity scale: (1) no problems, (2) slight problems, (3) moderate problems, (4) severe problems, and (5) extreme problems. The EQ VAS provides a quantitative measure of a patient’s overall perceived health, ranging from 0 to 100, with higher values indicating better health status.

For the purposes of this study, responses were dichotomized as ‘no problems’ for level 1 and as ‘problems’ for levels 2 to 5, corresponding to any degree of reported problems. This dichotomization strategy is recommended in EuroQol methodological guidance and is widely used in studies of diabetes and other chronic conditions to describe health-related quality of life and to model the presence or absence of problems within each domain. In addition, this approach enhances clinical interpretability and supports pragmatic decision-making in primary care settings.

### 2.4 Statistical analysis

Continuous variables were analyzed using the Kolmogorov-Smirnov test for normality and are presented as median (p25 – p75) due to their skewed distribution. Categorical variables are expressed as absolute and relative frequencies. Depending on the number of complications, differences among patients were observed using the Kruskal-Wallis test, Pearson’s chi-square test, and Fisher’s exact test for continuous and categorical variables, respectively. Differences between patients with and without problems in all dimensions were examined using the Mann–Whitney U test and Pearson’s Chi-square test.

The association between diabetes-related complications and HRQoL was determined using univariate and multivariate logistic regression analyses. Variables that showed a p-value <0.25 in the univariable analyses were identified as parameters potentially associated with HRQoL; therefore, they were included in the multivariable stepwise regression model and the model with the lowest Akaike Information Criterion (AIC) was selected. Multicollinearity among variables was assessed using the variance inflation factor with a threshold set at 10.

Euler diagrams (area-proportional diagrams) were used to visualize the overlap of diabetes-related complications using R with the ‘eulerr’ package.

Statistical significance was set at p<0.05. All analyses were conducted using R, version 3.5.3 (R Foundation for Statistical Computing, Vienna, Austria).

### 2.5 Handling of missing data

Before statistical analysis, all variables were thoroughly examined for missing data. For those variables with less than 10% missing values, a multivariate imputation by chained equations (MICE) method was applied using R version 3.5.3 (17). Subsequently, five independent imputed datasets were generated using the random forest algorithm, Regression models were fitted within each imputed dataset and pooled using Rubin’s rules to account for within- and between-imputation variability.

Sample size was calculated using G*Power, based on an effect size of 0.12 (18), a significance level (α) of 0.05, a statistical power of 0.80, and four predictors for multiple regression analysis.

## 3 Results

### 3.1 Clinical characteristics and factors associated with a cumulative presence of complications

A total of 1,703 patients were included in the DIABEMPIC program between 2017 and 2024. Of these, 26.3% showed no diabetes-related complications, 36.1% showed one complication, 23.8% showed two complications, and 13.6% showed three or more complications. The median (p25 – p75) age was 54 years (47 – 62). Most participants were women, and the majority had a family history of diabetes.

As shown in **Table 1**, the median age; diabetes duration increased progressively with the number of diabetes-related complications (p<0.001). Patients with a higher number of complications were also more likely to have hypertension. Likewise, younger age at diagnosis and lower EQ-VAS scores were observed among patients with multiple complications.

**Table 1:** Baseline characteristics of the patients classified according to the number of diabetes-related complications.

Variable	Total n = 1703	No complications n = 448	1 complication n = 616	2 complications n = 407	>3 complications n = 232	p-value
Age, years	54 (47 – 62)	51 (43 – 58.5)	53 (46 – 60)	56 (49 – 63)	59 (53 – 65)	**<0.001**
Sex, female	1021 (59.9)	277 (61.8)	369 (59.8)	246 (60.4)	129 (55.6)	0.47
BMI, kg/m^2^	28.8 (25.7 – 32.6)	29.4 (26.1 – 33.2)	28.9 (25.9 – 32.9)	28.6 (25.3 – 32.5)	27.9 (25.6 – 31.4)	**0.009**
Diabetes duration, years	10 (4 – 16)	5 (2 – 10)	8 (4 – 14)	14 (8 – 19)	17 (13 – 22)	**<0.001**
Age at diagnosis, years	44 (36 – 51)	45 (37 – 52)	44 (37 – 51)	43 (35 – 50)	41.5 (35 – 48)	**<0.001**
Family history of diabetes, n (%)	1494 (87.7)	385 (85.9)	543 (88.1)	352 (86.4)	214 (92.2)	0.09
Quality of life by VAS	60 (50 – 70)	70 (50 – 80)	60 (50 – 70)	50 (50 – 70)	50 (50 – 70)	**<0.001**
Educational attainment, n (%)						
Illiteracy	64 (3.7)	9 (2)	21 (3.4)	21 (5.1)	13 (5.6)	**0.001**
No education (can read and write)	133 (7.8)	27 (6)	48 (7.7)	37 (9)	21 (9)	
Primary	502 (29.4)	119 (26.5)	171 (27.7)	137 (33.6)	75 (32.3)	
Secondary	487 (28.5)	122 (27.2)	199 (32.3)	104 (25.5)	62 (26.7)	
High school	345 (20.2)	108 (24.1)	115 (18.6)	82 (20.1)	40 (17.2)	
University or more	172 (10)	63 (14)	62 (10)	26 (6.3)	21 (9)	
Smoking status, n (%)						
Current smoker	380 (22.3)	102 (21.7)	151 (24.5)	81 (19.9)	43 (18.5)	0.16
Former smoker	790 (46.3)	196 (42.7)	298 (48.3)	189 (46.4)	107 (46.1)	0.52
Comorbidities, n (%)						
Hypertension	823 (48.3)	177 (39.5)	258 (41.8)	223 (54.7)	165 (71.1)	**<0.001**
Hypertriglyceridemia	1069 (62.7)	269 (60)	398 (64.6)	250 (61.4)	152 (65.5)	0.33
Hypercholesterolemia	778 (45.6)	183 (40.8)	281 (45.6)	196 (48.1)	118 (50.8)	0.051
SBP, mmHg	120 (110 – 133)	120 (110 – 130)	120 (110 – 130)	121 (110 – 139)	130 (120 – 145.2)	**<0.001**
DBP, mmHg	74 (70 – 80)	75 (70 – 80)	72 (70 – 80)	75 (68 – 80)	76 (70 – 80)	0.12

BMI, body mass index; DPP4, Dipeptidyl peptidase 4 inhibitors; ACE inhibitor, angiotensin-converting enzyme inhibitors; ARB, angiotensin II receptor blockers. Data are expressed as medians (p25 – p75), or number (percentages) when corresponds.

Bold values indicate statistical significance (p < 0.05).

Moreover, serum creatinine, triglyceride, and cholesterol levels; and systolic blood pressure increased as the number of diabetes-related complications increased, showing statistically significant differences between the groups (p<0.001). Similarly, the proportion of patients with hypertension and those who were under insulin treatment, ACE inhibitors, thiazides, and ARBs also increased in accordance with the number of complications (**Supplementary Table 1**).

**Figure 1:**
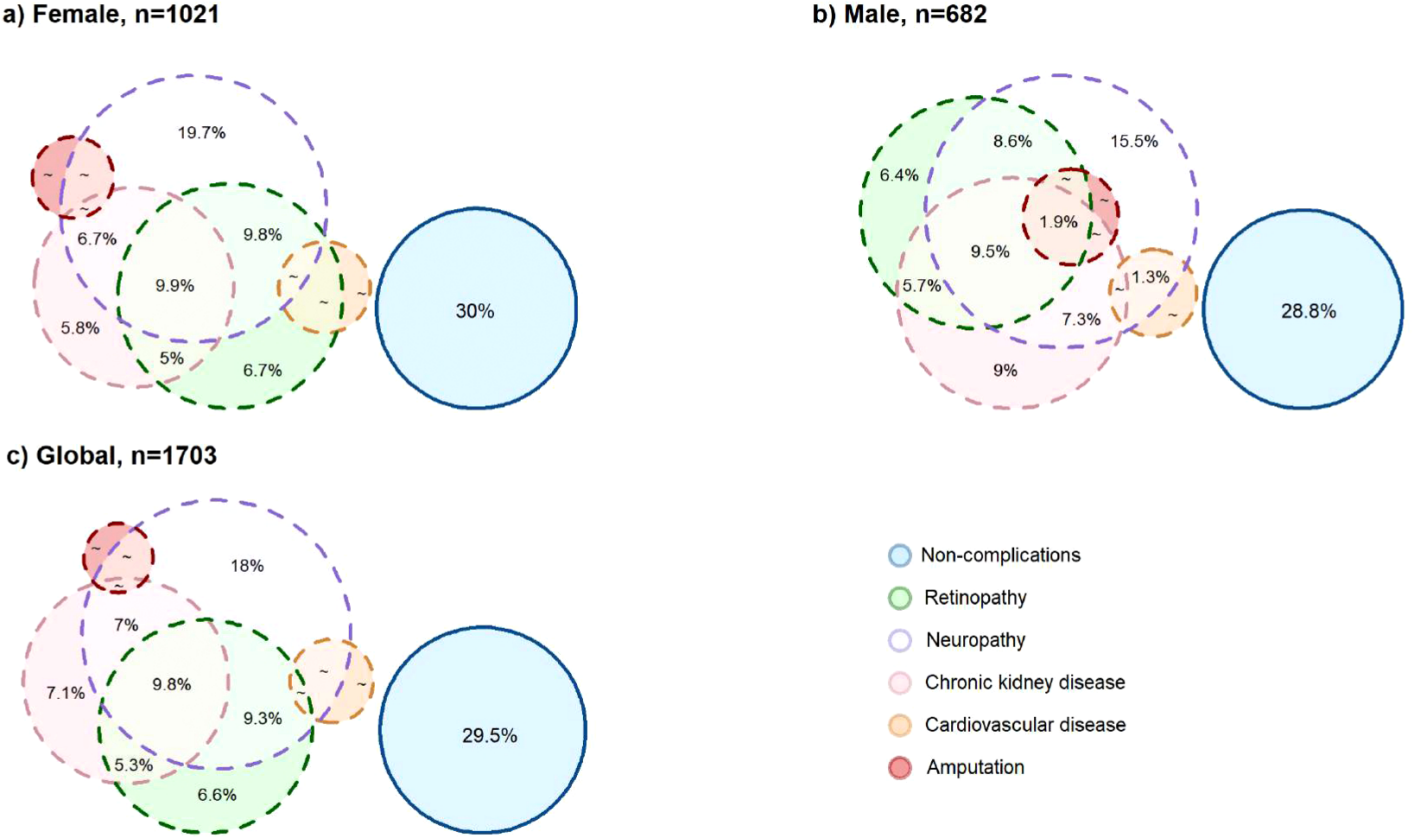
Area-proportional Euler diagram depicting the overlap of diabetes-related complications. Each circle represents the proportion of patients and the intersection of specific diabetes-related complications. Percentages are reported according to each stratum and the overall population. **(a)** Euler diagram illustrating the proportion of females with each diabetes-related complication and their intersections. **(b)** Euler diagram illustrating the proportion of males with each diabetes-related complication and their intersections. **(c)** Euler diagram illustrates the proportion of overall patients with each diabetes-related complication and their intersections. ~~ corresponds to proportion <1%.

As shown in **Figures 1a-c**, the Euler diagrams show that the most frequent diabetes-related complication was neuropathy (48.9%), followed by retinopathy (34.5%) and CKD (32.9%). Approximately 29.5% of the overall population had no documented complications.

Beyond single-complication prevalence, the Euler diagram showed substantial overlap between microvascular complications. The most predominant intersection was between neuropathy and CKD, followed by neuropathy and retinopathy (9.8% and 9.3%, respectively). Additionally, a notable proportion of patients presented triple involvement of neuropathy, CKD, and retinopathy (7%). Sex-stratified analysis showed similar overlap patterns across females and males. The prevalence of neuropathy in females was 49.6%, whereas in males was slightly lower (47.8%). Likewise, males showed a higher prevalence of CKD than comparison with females (37.3% vs. 29.9%, respectively), as well as for retinopathy (35.1% vs. 34.2%), CVD (5.8% vs. 4.9%), and amputation (3.2% vs. 0.8%). Globally, retinopathy was the second most prevalent complication (34.5%), followed by CKD (32.9%), CVD (5.3%), and a history of amputation (1.8%).

### 3.2 Differences in HRQoL responses according to complication status

The responses to the EQ-5D-5L are shown in **Supplementary Table 2**. Pain/discomfort and anxiety/depression had the highest prevalence of reported problems (53.8% and 53.4%, respectively), followed by mobility (34.2%), self-care (34%), and usual activities (24.2%). Across all five dimensions, the proportion of patients who responded “not having problems” was higher in the no-complications group. The group with complications showed the highest proportion in all the levels who referred a problem (“slight,” “moderate,” “severe,” and “unable”) for the three functional dimensions (mobility, self-care and usual activities). Similarly, for pain/discomfort and anxiety/depression dimensions, the group with complications showed the highest proportions in the levels “moderate,” “severe,” and “unable”.

### 3.3 Clinical characteristics and type of complication associated with impairment in specific HRQoL domains

As shown in **Table 2**, longer diabetes duration and being female were more frequent among patients who reported problems in all EQ-5D-5L dimensions (p<0.001). Additionally, in four of the five EQ-5D-5L dimensions, patients who reported problems were significantly older and had hypertension and hypercholesterolemia. Pharmacological treatment variables and laboratory parameters are detailed in **Supplementary Table 3**.

**Table 2:** Characteristics of the patients classified according to the problems reported in each of the five EQ-5D-5L dimensions.

	Mobility			Self-care			Usual activities			Pain/discomfort			Anxiety/depression		
Variable	No problems n = 1120	Problems n = 583	p-value	No problems n = 1123	Problems n = 580	p-value	No problems n = 1290	Problems n = 413	p-value	No problems n = 792	Problems n = 911	p-value	No problems n = 786	Problems n = 917	p-value
Age, years	53 (46 – 60)	57 (50.5 – 64)	**<0.001**	53 (46 – 60)	57 (50 – 64)	**<0.001**	54 (47 – 61)	56 (49.5 – 64)	**<0.001**	53 (45 – 60)	55 (48 – 62)	**<0.001**	55 (47 – 62)	54 (47 – 61)	0.60
Sex, female	635 (56.6)	386 (66.2)	**<0.001**	637 (56.8)	384 (66.2)	**<0.001**	756 (58.6)	265 (64.1)	**0.03**	427 (54)	594 (65.2)	**<0.001**	381 (48.4)	640 (69.7)	**<0.001**
BMI, kg/m^2^	28.4 (25.3 – 32)	29.5 (26.3 – 34)	**<0.001**	28.4 (25.3 – 31.8)	29.6 (26.3 – 34)	**<0.001**	28.7 (25.7 – 32.2)	29 (25.5 – 33.7)	0.10	28.5 (25.4 – 32.2)	29 (26 – 33)	**0.01**	28.1 (25.4 – 31.7)	29.3 (26 – 33.4)	**<0.001**
Diabetes duration, years	9 (3 – 15)	12 (6 – 18)	**<0.001**	9 (3 – 15)	12 (6 – 18)	**<0.001**	9 (3 – 15)	12 (6 – 18)	**<0.001**	8 (3 – 15)	11 (5 – 17)	**<0.001**	9 (4 – 15)	10 (4 – 17)	**<0.001**
Age at diagnosis, years	43 (35 – 50)	45 (36 – 52)	**0.01**	43 (36 – 50)	45 (36 – 52)	**0.04**	44 (36 – 50)	45 (36 – 51)	0.59	43 (36 – 51)	44 (36 – 50)	0.87	44 (36 – 52)	44 (35 – 50)	0.06
Family history of diabetes, n (%)	986 (88)	508 (87.1)	0.64	990 (88.1)	504 (86.8)	0.60	1134 (87.9)	360 (87.1)	0.77	692 (87.3)	802 (88)	0.74	679 (86.3)	815 (88.8)	0.17
Comorbidities, n (%)															
Hypertension	476 (42.5)	347 (59.5)	**<0.001**	480 (42.7)	343 (59.1)	**<0.001**	593 (45.9)	230 (55.6)	**<0.001**	365 (46)	458 (50.2)	0.13	352 (44.7)	471 (51.3)	**0.01**
Hypertriglyceridemia	691 (61.6)	378 (64.8)	0.23	696 (61.9)	373 (64.3)	0.39	807 (62.5)	262 (63.4)	0.63	485 (61.2)	584 (64.1)	0.23	484 (61.5)	585 (63.7)	0.40
Hypercholesterolemia	470 (41.9)	308 (52.8)	**<0.001**	469 (41.7)	309 (53.2)	**<0.001**	559 (43.3)	219 (53)	**0.001**	327 (41.2)	451 (49.5)	**0.001**	345 (43.8)	433 (47.2)	0.19
Complications related-diabetes, n (%)															
Peripheal neuropathy	461 (41.1)	372 (63.8)	**<0.001**	461 (41.1)	372 (63.8)	**<0.001**	569 (44.1)	264 (63.9)	**<0.001**	292 (36.8)	541 (59.3)	**<0.001**	324 (41.2)	509 (55.5)	**<0.001**
Chronic kidney disease	336 (30)	227 (38.9)	**<0.001**	340 (30.2)	223 (38.4)	**<0.001**	399 (30.9)	164 (39.7)	**0.001**	256 (32.3)	307 (33.6)	0.73	265 (33.7)	298 (32.4)	0.61
Diabetic retinopathy	338 (30.1)	251 (43)	**<0.001**	342 (30.4)	247 (42.5)	**<0.001**	414 (32)	175 (42.3)	**<0.001**	246 (31)	343 (37.6)	**0.005**	271 (34.4)	318 (34.6)	0.83
Amputation	11 (0.9)	20 (3.4)	**<0.001**	12 (1)	19 (3.2)	**0.002**	17 (1.3)	14 (3.3)	**0.01**	18 (2.2)	13 (1.4)	0.26	18 (2.2)	13 (1.4)	0.32
Cardiovascular disease	45 (4)	46 (7.8)	**0.001**	45 (4)	46 (7.8)	**0.001**	59 (4.5)	32 (7.7)	**0.01**	29 (3.6)	62 (6.8)	**0.005**	36 (4.5)	55 (5.9)	0.24
Non-complication	346 (30.8)	102 (17.4)	**<0.001**	345 (30.7)	103 (17.7)	**<0.001**	378 (29.3)	70 (16.9)	**<0.001**	256 (32.3)	192 (21)	**<0.001**	228 (29)	220 (23.9)	**<0.001**
1 complication	424 (37.8)	192 (32.5)		428 (38.1)	188 (32.4)		483 (37.4)	133 (32.2)		298 (37.6)	318 (34.9)		283 (36)	333 (36.3)	
2 complications	237 (21.1)	170 (29.5)		238 (21.1)	169 (29.1)		282 (21.8)	125 (30.2)		157 (19.8)	250 (27.4)		177 (22.5)	230 (25)	
≥3 complications	113 (10)	119 (20.4)		112 (10)	120 (20.6)		147 (11.3)	85 (20.5)		81 (10.2)	151 (16.5)		99 (12.5)	133 (14.5)	
SBPmmHg	120 (110 – 130)	125 (110 – 138)	**<0.001**	120 (110 – 130)	125 (110 – 138)	**<0.001**	120 (110 – 131.5)	120 (110 – 133.5)	0.97	120 (110 – 131)	121 (110 – 133)	0.17	120 (110 – 130)	122 (110 – 135)	**0.008**
DBP, mmHg	75 (70 – 80)	73 (69 – 80)	0.19	75 (70 – 80)	73 (69 – 80)	0.37	75 (70 – 80)	73 (69 – 80)	**0.01**	75 (70 – 80)	73 (70 – 80)	**0.03**	75 (70 – 80)	74 (69.7 – 80)	0.98

BMI: body mass index; DPP4: Dipeptidyl peptidase 4 inhibitors; ACE inhibitor: angiotensin-converting enzyme inhibitors; ARB: angiotensin II receptor blockers.

Data are expressed as medians (p25 – p75), or number (percentages) when corresponds.

Bold values indicate statistical significance (p < 0.05).

Regarding the number of diabetes-related complications, all EQ-5D-5L dimensions showed a statistically significantly higher proportion of patients reporting problems when two or more diabetes-related complications were present compared to those without reported problems. In the mobility, self-care, and usual activities dimensions, the single presence of any of the five diabetes-related complications was more common among patients reporting problems.

However, certain diabetes-related complications such as CKD and amputation history for the pain/discomfort dimension and CKD, diabetic retinopathy, amputation history, and CVD for the anxiety/depression dimension showed no difference with patients who reported problems. Nevertheless, peripheral neuropathy was the most prevalent in all EQ-5D-5L dimensions and showed being the only complication who affects the five dimensions (**Supplementary Table 4**).

Univariate analysis showed that being female, having a higher BMI, and a longer duration of diabetes were associated with an increased risk of reporting problems in any of the five dimensions. Likewise, older age, hypercholesterolemia, and an increasing number of complications were significantly associated with a higher likelihood of reporting problems across all dimensions, except the anxiety/depression dimension. Hypertension, in turn, showed a significant association with the risk of reporting problems in mobility, self-care, usual activities, and anxiety/depression dimensions.

Regarding the number of complications, all dimensions showed a higher proportion of patients who reported problems when two or more complications were present (**Supplementary Table 5**).

### 3.4 Independent factors associated with impairment in specific HRQoL domains

After adjusting for potential confounders, the multivariable logistic regression analysis (**Table 3**) demonstrated that female sex remained significantly associated with reporting problems in all HRQoL dimensions as well as the increasing risk of having problems as the number of complications increased in the three functional dimensions (mobility, self-care, and usual activities) and pain/discomfort. In the anxiety/depression dimension, risk increased, starting from the presence of two complications. Longer diabetes duration was independently associated with problems in the pain/discomfort dimension, and hypertension was also associated with HRQoL impairment in the functional domains (mobility, self-care, and usual activities). Likewise, the use of insulin, beta-blockers, statins, ACE inhibitors, bezafibrate, and acid acetylsalicylic acid was associated with a higher likelihood of HRQoL impairment. On the other hand, the use of DPP-4 inhibitors was the only protective factor against problems in the mobility and self-care dimensions (OR 0.53 [95%CI 0.39 – 0.72], p=0.001; OR 0.54 [95%CI 0.39 – 0.72], p=0.001; respectively).

**Table 3:** Multivariable logistic regression analysis for each dimension of the EQ-5D-5L.

Variable	Mobility OR (CI 95%)	p-value	Self- care OR (CI 95%)	p-value	Usual activities OR (CI 95%)	p-value	Pain/discomfort OR (CI 95%)	p-value	Anxiety/depression OR (CI 95%)	p-value
Sex, female	1.54 (1.23 – 1.92)	<0.001	1.57 (1.26 – 1.96)	<0.001	1.31 (1.04 – 1.67)	0.02	1.61 (1.32 – 1.98)	<0.001	2.44 (1.99 – 2.98)	**<0.001**
BMI, kg/m^2^									1.02 (1.007 – 1.04)	**0.004**
Diabetes duration, years	1 (1 – 1.02)	0.19	1 (1 – 1.02)	0.17	1 (1 – 1.02)	0.051	1.01 (1.002 – 1.02)	0.02		
Comorbidities										
Hypertension	1.63 (1.31 – 2.03)	<0.001	1.60 (1.29 – 2)	<0.001						
Diabetes-related complications										
Non-complications	*ref*	*ref*	*ref*	*ref*	*ref*	*Ref*	*ref*	*ref*	*ref*	*ref*
1 complication	1.46 (1.10 – 1.96)	0.009	1.43 (1.07 – 1.92)	0.01	1.46 (1.06 – 2.04)	0.02	1.34 (1.04 – 1.73)	0.02	1.22 (0.94 – 1.56)	0.12
2 complications	2.16 (1.56 – 2.98)	<0.001	2.11 (1.53 – 2.92)	<0.001	2.10 (1.48 – 3)	<0.001	1.77 (1.32 – 2.37)	<0.001	1.34 (0.98 – 1.73)	0.06
≥3 complications	3 (2.07 – 4.39)	<0.001	2.99 (2.06 – 4.37)	<0.001	2.72 (1.82 – 4)	<0.001	2.14 (1.5 – 3)	<0.001	1.52 (1.05 – 2)	**0.02**
Pharmacotherapy,										
Insulin	1.33 (1.05 – 1.68)	0.01	1.36 (1.07 – 1.72)	0.009	1.19 (0.92 – 1.53)	0.18	1.36 (1.03 – 1.58)	0.02		
DPP-4 inhibitors	0.53 (0.39 – 0.71)	<0.001	0.53 (0.39 – 0.72)	<0.001	0.72 (0.51 – 0.98)	0.04				
ACE inhibitor									1.48 (1.12 – 1.98)	**0.01**
Beta-blockers										
Statins	1.67 (1.27 – 2.20)	<0.001	1.69 (1.28 – 2.23)	<0.001			1.53 (1.18 – 2)	0.001		
Bezafibrate									1.49 (1.12 – 2)	**0.01**
Acetylsalicylic Acid					1.70 (1.14 – 2.51)	0.007				

BMI, body mass index; DPP4, Dipeptidyl peptidase 4 inhibitors; ACE inhibitor, angiotensin-converting enzyme inhibitors; ARB, angiotensin II receptor blockers.

Bold values indicate statistical significance (p < 0.05).

## 4 Discussion

This study aimed to assess the impact of diabetes-related complications on HRQoL in individuals with T2D receiving primary care. To our knowledge, this is the largest study to date in the Latin American region to examine the effect of diabetes-related complications on specific HRQoL domains, based on updated and systematically collected data. Our findings show that a higher number of complications is associated with progressively greater, domain-specific impairment in quality of life. Notably, HRQoL deterioration was evident from the presence of even a single complication, highlighting the importance of prevention and early identification, particularly in primary care settings, where timely interventions remain feasible. In addition, easily identifiable characteristics such as female sex and the presence of hypertension were independently associated with HRQoL impairment, reinforcing their practical relevance for risk stratification and targeted management in routine diabetes care.

Our study contributes to our understanding of the burden of diabetes-related complications by illustrating their frequent coexistence and interplay. In Mexico and other low- and middle-income countries, knowledge about complication prevalence comes from studies with self-reported data, estimations derived from indirect data, or from registries and health records where detection of complications is inconsistently performed. In this context, our study revealed that when complications were actively screened, a substantial proportion of individuals with type 2 diabetes in primary care were found to have clusters of complications. For example, up to 65% of individuals with retinopathy also present with peripheral neuropathy and nearly 80% of those with CKD have at least one additional complication. Managing multimorbidity is becoming an increasing global challenge and a key concern for health systems, with T2D emerging as a major contributor and a central driver of multiple long-term conditions (19, 20). In this context, beyond the cumulative burden of diabetes-related complications, a substantial proportion of individuals in our study also presented with coexisting chronic conditions, further highlighting the complexity of care needs in this population. The results of our study provide valuable insights to guide organizational strategies aimed at implementing integrated care approaches for individuals with multiple long-term conditions.

Our findings highlight important considerations for advancing person-centered diabetes care. Traditionally, clinical and policy efforts have emphasized the prevention of advanced complications (e.g., myocardial infarction and kidney disease requiring substitutive therapy), as the main strategy to preserve HRQoL. However, this approach often overlooks the substantial impairment in HRQoL caused by diabetes-related complications long before major clinical outcomes occur. Our analysis showed that even early and frequently coexisting complications, such as neuropathy, retinopathy, and CKD, are associated with significant deterioration across multiple HRQoL domains. Notably, these same complications represent the primary contributors to the lethal and non-lethal burden of disease in Mexico, according to the most recent Global Burden of Disease report (21, 22). These considerations support the need to shift toward a more comprehensive, anticipatory, and quality-of-life-centric approach to diabetes care. Moreover, the higher prevalence of complications and their combinations among men, along with the disproportionately greater impact on HRQoL observed in women, highlights the need for sex-sensitive approaches in diabetes care. Tailoring interventions to address sex-specific vulnerabilities may help reduce health disparities and enhance outcomes in men and women with T2D.

The associations observed between several pharmacological therapies and lower HRQoL are likely influenced by confounding by indication and underlying disease severity. Therefore, these findings should not be interpreted as evidence of a detrimental effect of these treatments on quality of life.

In Latin America, evidence on the cumulative impact of diabetes-related complications in primary care settings remains limited. A systematic review by De la Cruz et al. reported that complications such as neuropathy, nephropathy, cardiopathy, and diabetic foot negatively affect the quality of life (7). To our knowledge, this study is the first in the region to evaluate the cumulative burden of complications and their domain-specific impact on HRQoL. Our findings are consistent with those reported by Sullivan et al. in a large multi-country analysis, showing that the impact of diabetes-related complications on HRQoL is cumulative and non-linear, with distinct effects across complications (23).

In this context, we observed stronger associations between cumulative complications and impairment in functional dimensions such as mobility, self-care, and usual activities, while pain/discomfort and anxiety/depression were also consistently affected to a lesser extent. In this regard, when complications were analyzed individually, most did not show a significant impact on the anxiety/depression domain. This suggests that emotional distress in our population may be driven by d potentially modifiable factors beyond isolated diabetes complications.

The incorporation of patient-reported outcomes, including HRQoL, into both research and routine diabetes care has gained increasing recognition in recent years (3, 24). Assessing HRQoL can help healthcare providers identify individuals with specific needs that may hinder the achievement of care goals, as lower HRQoL has been associated with poor treatment adherence, reduced social support, emotional distress, suboptimal self-management, low self-efficacy, and socioeconomic disadvantages (25–27). In our study, the cumulative burden of complications emerged as a strong factor associated with HRQoL impairment, suggesting the value of systematically assessing HRQoL, particularly in patients at early stages of complications. This approach may enhance clinical practice by improving communication, promoting behavioral change, facilitating the identification of high-risk patients, and enabling targeted and person-centered interventions to preserve health and quality of life.

Improving HRQoL is a feasible and meaningful goal even for individuals already living with diabetes-related complications. Adopting a quality-of-life-centric perspective allows healthcare systems to address two complementary objectives: enhancing the current well-being of patients and implementing evidence-based strategies to prevent future deterioration. From a health system standpoint, multicomponent quality improvement strategies that incorporate patient education, self-management support, case management, registries, and team-based care have been shown to improve clinical outcomes and, consequently, HRQoL (12, 28). As quality of life represents the ultimate goal of health interventions, these findings to underscore the need to strengthen primary care systems to preserve or improve health through proactive prevention, early detection, and integration of multicomponent care strategies tailored to the needs of people living with diabetes.

Future research should incorporate longitudinal designs to better characterize temporal relationships between diabetes-related complications and HRQoL trajectories. In addition, the integration of standardized measures of psychological distress, social vulnerability, and treatment adherence may help clarify the mechanisms underlying HRQoL impairment in primary care populations. Implementation studies are also needed to evaluate whether systematic HRQoL assessment can inform targeted interventions and improve patient-centered outcomes in routine care.

A key strength of this study is the prospective collection of clinical data using standardized methodologies as well as the inclusion of a large sample drawn from multiple primary healthcare units. However, this study had several limitations. First, its cross-sectional design precludes the establishment of causal relationships. Second, although the study included a large primary care population, the participants were recruited from urban areas and the public healthcare system, where adverse social determinants of health are common. This might limit the generalizability of our findings to other populations. Nonetheless, this group represents the majority of individuals receiving care in Mexico and likely reflects similar contexts in other Latin American countries. Third, this study did not assess the severity of individual complications which may have led to an underestimation of the impact of advanced disease on HRQOL. However, the primary aim was to evaluate quality of life in typical primary care settings, where non-advanced complications are most prevalent, enhancing the relevance of our findings. In addition, dichotomization of EQ-5D-5L responses may have reduced information on severity gradients. Nevertheless, this approach is consistent with methodological recommendations and supports clinically meaningful interpretation in primary care. Fourth, we did not include other potentially important factors, such as mental health disorders, diabetes-related emotional distress, social support, and health literacy, which may also influence HRQoL and help explain some of the observed associations.

In particular, the absence of these measures may have contributed to residual confounding in the anxiety/depression domain, and therefore findings related to this dimension should be interpreted with caution.

## 5 Conclusions

In summary, we observed a high prevalence and coexistence of diabetes-related complications among individuals receiving conventional care in primary care settings in Mexico, which had a substantial impact on HRQoL. The cumulative burden of complications, female sex, and hypertension emerged as strong factors associated with HRQoL impairment, with differentiated effects across specific domains. While anxiety/depression and pain/discomfort were the most frequently reported affected dimensions, the accumulation of complications was more strongly associated with impairments in functional domains, such as mobility, self-care, and usual activities.

These findings advocate the implementation of quality-of-life-centric strategies in the management of type 2 diabetes, particularly within primary care systems in low-income and middle-income countries. The early impact and frequent coexistence of complications underscore the need for timely identification and comprehensive care aimed at preventing their onset and managing their progression. As the growing demand for healthcare systems has increasingly shifted the management of patients with diabetes-related complications to the primary care level, there is a critical need for policy action and strategic resource allocation to strengthen primary health systems in the Latin American region. Implementing multicomponent interventions in these settings may enhance both clinical and functional outcomes, supporting more equitable, person-centered care, and helping to mitigate the growing burden of diabetes in the region.

## Data Availability

The raw data supporting the conclusions of this article will be made available by the authors, without undue reservation.
