# Supplementary material for: Cumulative burden of diabetes-related complications and health-related quality of life in primary care: a cross-sectional study from Mexico
Source: Front Clin Diabetes Healthc. 2026 Mar 12;7:1773692. doi: 10.3389/fcdhc.2026.1773692 (PMC13019879; doi:10.3389/fcdhc.2026.1773692)
Supplement: Supplementary file 1 [file DataSheet1.docx]

**Supplementary Material: Cumulative burden of diabetes-related complications and health-related quality of life in primary care: a cross-sectional study from Mexico**

**Supplementary Table S1:** Baseline characteristics of the patients classified according to the number of diabetes-related complications

| Variable | Total  n = 1703 | No complications  n = 448 | 1 complication  n = 616 | 2 complications  n = 407 | >3 complications  n = 232 | p-value |
| --- | --- | --- | --- | --- | --- | --- |
| Pharmacotherapy, n (%) |  |  |  |  |  |  |
| Metformin | 1380 (81) | 360 (80.3) | 513 (83.2) | 334 (82) | 173 (74.5) | **0.03** |
| Insulin | 1004 (59) | 199 (44.4) | 331 (53.7) | 293 (72.3) | 181 (79.7) | **<0.001** |
| DPP-4 inhibitors | 282 (16.5) | 63 (14) | 104 (16.8) | 77 (18.9) | 38 (16.3) | 0.29 |
| ACE inhibitor | 246 (14.4) | 49 (10.9) | 83 (13.4) | 72 (17.6) | 42 (18.1) | **0.01** |
| Thiazides | 120 (7) | 22 (4.9) | 38 (6.1) | 36 (8.8) | 24 (10.3) | **0.02** |
| ARB | 403 (23.6) | 85 (18.9) | 125 (20.2) | 108 (26.5) | 85 (36.6) | **<0.001** |
| Beta-blockers | 82 (4.8) | 12 (2.6) | 25 (4) | 29 (7.1) | 16 (6.8) | **0.006** |
| Statins | 283 (16.6) | 69 (15.4) | 108 (17.5) | 77 (18.9) | 29 (12.5) | 0.15 |
| Bezafibrate | 237 (13.9) | 58 (12.8) | 89 (14.4) | 69 (17) | 21 (9.3) | **0.01** |
| Acetylsalicylic Acid | 127 (7.4) | 27 (6) | 40 (6.4) | 41 (10) | 19 (8.1) | **0.09** |
| Laboratories |  |  |  |  |  |  |
| HbA1c, % | 9.5 (7.9 – 11.2) | 9 (7.5 – 11) | 9.7 (8 – 11.3) | 9.5 (8 – 11.3) | 9.5 (8.4 – 11.4) | **0.009** |
| Serum cretinine, mg/dl | 0.8 (0.6 – 0.9) | 0.7 (0.6 – 0.8) | 0.7 (0.6 – 0.9) | 0.8 (0.6 – 1) | 0.9 (0.7 – 1.2) | **<0.001** |
| Tryglycerides, mg/dl | 174 (121.8 – 258) | 166 (117 – 253) | 180 (128 – 266) | 169 (117 – 242) | 184.5 (130 – 270) | 0.05 |
| Cholesterol, mg/dl | 186 (160 – 215) | 181.5 (158 – 211) | 186 (160 – 214.5) | 188.5 (161 – 220) | 191 (164 – 230) | **0.04** |
|  |  |  |  |  |  |  |

BMI: body mass index; DPP4: Dipeptidyl peptidase 4 inhibitors; ACE inhibitor: angiotensin-converting enzyme inhibitors; ARB: angiotensin II receptor blockers. Data are expressed as medians (p25 – p75),

or number (percentages) when corresponds.

|  | **Mobility** | | **Self-care** | | **Usual activities** | | **Pain/discomfort** | | **Anxiety/depression** | |
| --- | --- | --- | --- | --- | --- | --- | --- | --- | --- | --- |
| Level of problems | No complications  n= 448 | Complications  n= 1255 | No complications  n= 448 | Complications  n= 1255 | No complications  n= 448 | Complications  n= 1255 | No complications  n= 448 | Complications  n= 1255 | No complications  n= 448 | Complications  n= 1255 |
| No problems, n (%) | 346 (77.2) | 774 (61.6) | 345 (77) | 778 (61.9) | 378 (84.3) | 912 (72.6) | 256 (57.1) | 536 (42.7) | 228 (50.8) | 559 (44.5) |
| Slight problems, n (%) | 57 (12.7) | 232 (18.4) | 59 (13.1) | 230 (18.3) | 43 (9.5) | 185 (14.7) | 109 (24.3) | 296 (23.5) | 112 (25) | 287 (22.8) |
| Moderate problems, n (%) | 35 (7.8) | 162 (12.9) | 35 (7.8) | 162 (12.9) | 17 (3.7) | 95 (7.5) | 59 (13.1) | 223 (17.7) | 59 (13.1) | 227 (18) |
| Severe problems, n (%) | 9 (2) | 80 (6.3) | 8 (1.7) | 78 (6.2) | 9 (2) | 50 (3.9) | 18 (4) | 176 (14) | 37 (8.2) | 124 (9.8) |
| Unable, n (%) | 1 (0.2) | 7 (0.5) | 1 (0.2) | 7 (0.5) | 1 (0.2) | 13 (1) | 6 (1.3) | 24 (1.9) | 12 (2.6) | 58 (4.6) |
| p-value | <0.001 |  | <0.001 |  | <0.001 |  | <0.001 |  | 0.01 |  |

**Supplementary Table S2: Health-related quality of life dimensions according to EQ-5D and by number of complications**

Data are expressed as number (percentages).

**Supplementary Table S3:** Characteristics of the patients classified according to the problems reported in each of the five EQ-5D-5L dimensions

|  | **Mobility** |  |  | **Self-care** |  |  | **Usual activities** |  |  |
| --- | --- | --- | --- | --- | --- | --- | --- | --- | --- |
|  | **No problems**  **n = 1120** | **Problems**  **n = 583** | **p-value** | **No problems**  **n = 1123** | **Problems**  **n = 580** | **p-value** | **No problems**  **n = 1290** | **Problems**  **n = 413** | **p-value** |
| **Pharmacotherapy, n (%)** | |  |  |  |  |  |  |  |  |
| Metformin | 896 (80) | 484 (83) | 0.12 | 899 (80) | 481 (83.1) | 0.14 | 1031 (79.9) | 349 (84.5) | **0.03** |
| Insulin | 604 (53.9) | 397 (68) | **<0.001** | 605 (53.8) | 396 (68.2) | **<0.001** | 724 (56.1) | 277 (67) | **<0.001** |
| DPP-4 inhibitors | 211 (18.8) | 71 (12.1) | **<0.001** | 212 (18.8) | 70 (12) | **<0.001** | 225 (17.4) | 57 (13.8) | 0.08 |
| ACE inhibitor | 143 (12.7) | 103 (17.6) | **0.007** | 144 (12.8) | 102 (17.5) | **0.009** | 171 (13.2) | 75 (18.1) | **0.01** |
| Thiazides | 75 (6.6) | 45 (7.7) | 0.49 | 75 (6.6) | 45 (7.7) | 0.46 | 85 (6.5) | 35 (8.4) | 0.23 |
| ARB | 235 (20.9) | 168 (28.8) | **<0.001** | 235 (20.9) | 168 (28.9) | **<0.001** | 289 (22.4) | 114 (27.6) | **0.04** |
| Beta-blockers | 36 (3.2) | 46 (7.8) | **<0.001** | 36 (3.2) | 46 (7.8) | **<0.001** | 56 (4.3) | 25 (6) | 0.17 |
| Statins | 161 (14.3) | 122 (20.9) | **<0.001** | 162 (14.4) | 121 (20.8) | **<0.001** | 200 (15.5) | 83 (20) | **0.04** |
| Bezafibrate | 146 (13) | 88 (15) | 0.27 | 147 (13) | 87 (15) | 0.31 | 176 (13.6) | 58 (14) | 0.90 |
| Acetylsalicylic Acid | 67 (5.9) | 60 (10.2) | **0.001** | 66 (5.8) | 61 (10.5) | **0.001** | 80 (6.2) | 47 (11.3) | **0.001** |
| **Laboratories** |  |  |  |  |  |  |  |  |  |
| HbA1c, % | 9.5 (7.9 – 11.2) | 9.5 (8 – 11.2) | 0.69 | 9.5 (7.8 – 11.2) | 9.6 (8.1 – 11.3) | 0.86 | 9.5 (7.9 – 11.2) | 9.5 (7.7 – 11.4) | 0.53 |
| Serum cretinine, mg/dl | 0.7 (0.6 – 0.9) | 0.7 (0.6 – 0.9) | 0.68 | 0.7 (0.6 – 0.9) | 0.7 (0.6 – 0.9) | 0.68 | 0.7 (0.6 – 0.9) | 0.7 (0.6 – 0.9) | 0.79 |
| Tryglycerides, mg/dl | 175 (120 – 260) | 172 (127 – 252) | 0.86 | 175 (119 – 260) | 172 (127 – 253) | 0.85 | 175 (122.5 – 265.7) | 173 (123.5 – 248) | 0.66 |
| Cholesterol, mg/dl | 182 (158 – 212) | 193 (164 – 224) | **<0.001** | 182 (158 – 212) | 194 (164 – 224) | **<0.001** | 184 (158 – 214) | 193 (166 – 223) | **0.001** |
|  |  |  |  |  |  |  |  |  |  |

BMI: body mass index; DPP4: Dipeptidyl peptidase 4 inhibitors; ACE inhibitor: angiotensin-converting enzyme inhibitors; ARB: angiotensin II receptor blockers. Data are expressed as medians (p25 – p75), or number (percentages) when corresponds.

**Supplementary Table S3:** Continued

|  | **Pain/discomfort** |  |  |  | **Anxiety/depression** | |  |
| --- | --- | --- | --- | --- | --- | --- | --- |
|  | **No problems**  **n = 792** | **Problems**  **n = 911** | **p-value** |  | **No problems**  **n = 786** | **Problems**  **n = 917** | **p-value** |
| **Pharmacotherapy, n (%)** | |  |  |  |  |  |  |
| Metformin | 624 (78.7) | 756 (82.9) | **0.03** |  | 625 (79.5) | 755 (82.3) | 0.20 |
| Insulin | 412 (52) | 589 (64.6) | **<0.001** |  | 424 (53.9) | 577 (62.9) | **<0.001** |
| DPP-4 inhibitors | 139 (17.5) | 143 (15.6) | 0.35 |  | 146 (18.5) | 136 (14.8) | **0.03** |
| ACE inhibitor | 98 (12.3) | 148 (16.2) | **0.02** |  | 94 (11.9) | 152 (16.5) | **0.007** |
| Thiazides | 56 (7) | 64 (7) | 0.99 |  | 56 (7.1) | 64 (6.9) | 0.99 |
| ARB | 189 (23.8) | 214 (23.4) | 0.98 |  | 181 (23) | 222 (24.2) | 0.68 |
| Beta-blockers | 28 (3.5) | 52 (5.7) | **0.04** |  | 33 (4.1) | 47 (5.1) | 0.43 |
| Statins | 107 (13.5) | 176 (19.3) | **0.001** |  | 118 (15) | 165 (17.9) | 0.13 |
| Bezafibrate | 90 (11.3) | 144 (15.8) | **0.006** |  | 89 (11.3) | 145 (15.8) | **0.009** |
| Acetylsalicylic Acid | 48 (6) | 79 (8.6) | **0.03** |  | 52 (6.6) | 75 (8.1) | 0.21 |
| **Laboratories** |  |  |  |  |  |  |  |
| HbA1c, % | 9.5 (7.8 – 11.3) | 9.6 (8.1 – 11.2) | 0.55 |  | 9.5 (7.8 – 11.2) | 9.5 (8 -11.3) | 0.78 |
| Serum cretinine, mg/dl | 0.8 (0.6 – 0.9) | 0.7 (0.6 – 0.9) | 0.10 |  | 0.8 (0.6 – 0.9) | 0.7 (0.6 – 0.9) | **0.001** |
| Tryglycerides, mg/dl | 175 (120 – 270) | 174 (125 – 249) | 0.85 |  | 174 (120 – 261) | 174 (124 – 254) | 0.82 |
| Cholesterol, mg/dl | 182 (157 – 211) | 190 (163 – 219) | **<0.001** |  | 184 (158 – 214) | 188 (162 – 217) | 0.08 |
|  |  |  |  |  |  |  |  |

.

.

BMI: body mass index; DPP4: Dipeptidyl peptidase 4 inhibitors; ACE inhibitor: angiotensin-converting enzyme inhibitors; ARB: angiotensin II receptor blockers.

Data are expressed as medians (p25 – p75), or number (percentages) when corresponds

**Supplementary Table S4: Univariable logistic regression analysis of diabetes-related complications according to each dimension of the EQ-5D-5L**

|  | **Mobility** |  | **Self- care** |  | **Usual activities** |  | **Pain/discomfort** |  | **Anxiety/depression** |  |
| --- | --- | --- | --- | --- | --- | --- | --- | --- | --- | --- |
|  | **OR (95% CI)** | **p-value** | **OR (95% CI)** | **p-value** | **OR (95% CI)** | **p-value** | **OR (95% CI)** | **p-value** | **OR (95% CI)** | **p-value** |
| Diabetic retinopathy | 1.66 (1.33 – 2) | <0.001 | 1.59 (1.27 – 1.97) | <0.001 | 1.5 (1.18 – 1.9) | <0.001 | 1.31 (1.06 – 1.63) | 0.01 | 1.04 (0.84 – 1.29) | 0.67 |
| Peripheal neuropathy | 2.56 (2 – 3.15) | <0.001 | 2.6 (2.11 – 3.2) | <0.001 | 2.26 (1.80 – 2.85) | <0.001 | 2.52 (2 – 3) | <0.001 | 1.82 (1.50 – 2.21) | **<0.001** |
| Chronic kidney disease | 1.55 (1.25 – 1.92) | <0.001 | 1.47 (1.19 -1.83) | <0.001 | 1.5 (1.19 – 1.9) | <0.001 | 1 (0.86 – 1.29) | 0.59 | 0.92 (0.75 – 1.13) | 0.44 |
| Cardiovascular disease | 1.96 (1.28 – 3) | 0.001 | 2 (1.30 – 3) | 0.001 | 1.76 (1.11 – 2.73) | 0.01 | 1.98 (1.27 – 3.17) | 0.003 | 1.29 (0.84 – 2) | 0.24 |
| Amputation | 3.59 (1.69 – 8.1) | 0.001 | 3.14 (1.49 – 6.91) | 0.003 | 2.86 (1.35 – 6) | 0.005 | 0.58 (0.27 – 1.22) | 0.15 | 0.49 (0.22 – 1.04) | 0.06 |

BMI: body mass index; DPP4: Dipeptidyl peptidase 4 inhibitors; ACE inhibitor: angiotensin-converting enzyme inhibitors; ARB: angiotensin II receptor blockers.

**Supplementary Table S5:** Univariable logistic regression analysis for each dimension of the EQ-5D

|  | **Mobility** |  | **Self- care** |  | **Usual activities** |  | **Pain/discomfort** |  | **Anxiety/depression** |  |
| --- | --- | --- | --- | --- | --- | --- | --- | --- | --- | --- |
|  | **OR (CI 95%)** | **p-value** | **OR (CI 95%)** | **p-value** | **OR (CI 95%)** | **p-value** | **OR (CI 95%)** | **p-value** | **OR (CI 95%)** | **p-value** |
| Age, years | 1.03 (1.02 – 1.04) | **<0.001** | 1.03 (1.02 – 1.04) | **<0.001** | 1.02 (1.01 – 1.03) | **<0.001** | 1.02 (1.01 – 1.03) | **<0.001** | 0.99 (0.99 – 1) | 0.69 |
| Sex, female | 1.52 (1.23 – 1.88) | **<0.001** | 1.52 (1.23 – 1.88) | **<0.001** | 1.29 (1.02 – 1.63) | **0.02** | 1.59 (1.31 – 1.94) | **<0.001** | 2.4 (1.96 – 2.93) | **<0.001** |
| BMI, kg/m^2^ | 1.03 (1.02 – 1.05) | **<0.001** | 1.04 (1.02 – 1.05) | **<0.001** | 1.01 (1 – 1.03) | 0.05 | 1.02 (1 – 1.03) | **0.01** | 1.03 (1.01 – 1.05) | **<0.001** |
| Diabetes duration, years | 1.03 (1.02 – 1.05) | **<0.001** | 1.03 (1.02 – 1.05) | **<0.001** | 1.03 (1.02 – 1.05) | **<0.001** | 1.03 (1.02 – 1.05) | **<0.001** | 1.01 (1 – 1.02) | **0.006** |
| Age at diagnosis, years | 0.99 (0.99 – 1) | 0.96 | 1 (0.99 – 1) | 0.90 | 0.99 (0.99 – 1) | 0.71 | 0.99 (0.99 - 1) | 0.45 | 0.99 (0.98 – 1) | **0.04** |
| **Comorbidities** |  |  |  |  |  |  |  |  |  |  |
| Hypertension | 2 (1.66 – 2.49) | **<0.001** | 2 (1.63 – 2.46) | **<0.001** | 1.49 (1.19 – 1.87) | **<0.001** | 1.20 (0.99 – 1.45) | 0.055 | 1.29 (1.06 – 1.56) | **0.008** |
| Hypertriglyceridemia | 1.11 (0.9 – 1.36) | 0.32 | 1.11 (0.9 – 1.38) | 0.29 | 1.05 (0.83 – 1.32) | 0.66 | 1.13 (0.93 – 1.38) | 0.19 | 1.08 (0.88 – 1.31) | 0.43 |
| Hypercholesterolemia | 1.54 (1.26 – 1.89) | **<0.001** | 1.58 (1.29 – 1.94) | **<0.001** | 1.43 (1.14 – 1.79) | **0.001** | 1.36 (1.12 – 1.65) | **0.001** | 1.12 (0.93 – 1.36) | 0.21 |
| **Diabetes-related complications** | |  |  |  |  |  |  |  |  |  |
| Non-complications | *ref* | *ref* | *ref* | *ref* | *ref* | *ref* | *ref* | *ref* | *ref* | *ref* |
| 1 complication | 1.5 (1.12 – 2) | **0.002** | 1.5 (1.12 – 2) | **0.004** | 1.53 (1.10 – 2.13) | **0.009** | 1.43 (1.03 – 1.83) | **0.004** | 1.18 (0.91 – 1.52) | 0.09 |
| 2 complications | 2.51 (1.85 – 3.41) | **<0.001** | 2.54 (1.89 – 3.44) | **<0.001** | 2.46 (1.75 – 3.46) | **<0.001** | 2 (1.57 – 2.79) | **<0.001** | 1.34 (1.01 – 1.78) | **0.03** |
| ≥3 complications | 3.90 (2.78 – 5.49) | **<0.001** | 3.82 (2.77 – 5.44) | **<0.001** | 3.22 (2.25 – 4.72) | **<0.001** | 2.57 (1.86 – 3.39) | **<0.001** | 1.41 (1.04 – 1.97) | **0.02** |
| **Pharmacotherapy** |  |  |  |  |  |  |  |  |  |  |
| Metformin | 1.28 (0.98 – 1.67) | 0.09 | 1.28 (0.98 – 1.68) | 0.06 | 1.41 (1.04 – 1.91) | **0.02** | 1.32 (1.03 – 1.7) | **0.01** | 1.17 (0.91 – 1.49) | 0.20 |
| Insulin | 1.83 (1.48 – 2.62) | **<0.001** | 1.85 (1.50 – 2.29) | **<0.001** | 1.58 (1.25 – 2) | **<0.001** | 1.68 (1.38 – 2) | **<0.001** | 1.41 (1.16 – 1.71) | **0.001** |
| DPP-4 inhibitors | 0.60 (0.45 – 0.8) | **0.001** | 0.59 (0.44 – 0.8) | **0.001** | 0.75 (0.55 – 1.03) | 0.07 | 0.87 (0.67 – 1.13) | 0.30 | 0.77 (0.6 – 1) | 0.05 |
| ACE inhibitor | 1.46 (1.11 – 1.94) | **0.007** | 1.49 (1.13 – 1.97) | **0.005** | 1.49 (1.10 – 2) | **0.009** | 1.42 (1.07 – 1.87) | **0.01** | 1.49 (1.13 – 1.98) | **0.005** |
| ARB | 1.54 (1.22 – 1.94) | **<0.001** | 1.54 (1.22 – 1.95) | **<0.001** | 1.32 (1.02 – 1.7) | **0.03** | 0.97 (0.77 – 1.21) | 0.80 | 1.07 (0.85 – 1.34) | 0.53 |
| Beta-blockers | 2.54 (1.61 – 4) | **<0.001** | 2.58 (1.63 – 4) | **<0.001** | 1.38 (0.84 – 2.25) | 0.20 | 1.63 (1.01 – 2.61) | **0.04** | 1.19 (0.75 – 1.89) | 0.43 |
| Statins | 1.62 (1.25 – 2.11) | **<0.001** | 1.62 (1.25 – 2.11) | **<0.001** | 1.39 (1.04 – 1.85) | **0.02** | 1.53 (1.18 – 2) | **0.001** | 1.24 (0.96 – 1.62) | 0.09 |
| Bezafibrate | 1.20 (0.90 – 1.6) | 0.19 | 1.21 (0.91 – 1.61) | 0.17 | 1.06 (0.76 – 1.45) | 0.70 | 1.48 (1.12 – 1.97) | **0.006** | 1.44 (1.09 – 1.92) | **0.01** |
| Acetylsalicylic Acid | 1.84 (1.27 – 2.66) | **0.001** | 1.87 (1.29 – 2.7) | **0.001** | 1.87 (1.27 – 2.74) | **0.001** | 1.47 (1.01 – 2.14) | **0.04** | 1.23 (0.85 – 1.79) | 0.26 |
| **Laboratories** |  |  |  |  |  |  |  |  |  |  |
| HbA1c, % | 0.99 (0.96 – 1.02) | 0.56 | 0.99 (0.96 – 1.02) | 0.57 | 0.98 (0.94 – 1.01) | 0.36 | 0.99 (0.97 – 1.02) | 0.96 | 1.002 (0.97 – 1.03) | 0.81 |
| Cholesterol, mg/dl | 1.006 (1.001 – 1.06) | **<0.001** | 1.003 (1.001 – 1.05) | **<0.001** | 1.003 (1.001 – 1.06) | **0.001** | 1.002 (1 – 1.004) | **0.004** | 1.002 (1 – 1.04) | **0.04** |
| SBP, mmHg | 1.009 (1.003 – 1.01) | **<0.001** | 1.009 (1.004 – 1.01) | **<0.001** | 0.99 (0.99 – 1.05) | 0.94 | 1.002 (0.99 – 1.07) | 0.40 | 1.006 (1.001 – 1.01) | **0.009** |

BMI: body mass index; DPP4: Dipeptidyl peptidase 4 inhibitors; ACE inhibitor: angiotensin-converting enzyme inhibitors; ARB: angiotensin II receptor blockers.
